# Correlation between High Temperature Deformation and β Relaxation in LaCe-Based Metallic Glass

**DOI:** 10.3390/ma13040833

**Published:** 2020-02-12

**Authors:** Yinghong Chen, Jichao Qiao

**Affiliations:** School of Mechanics, Civil Engineering and Architecture, Northwestern Polytechnical University, Xi’an 710072, China; cyhongc@mail.nwpu.edu.cn

**Keywords:** metallic glass, β relaxation, viscosity, Newtonian flow, activation volume

## Abstract

High-temperature deformation around the glass transition temperature T_g_ and the dynamic mechanical behavior of La_30_Ce_30_Al_15_Co_25_ metallic glass were investigated. According to dynamic mechanical analysis (DMA) results, La_30_Ce_30_Al_15_Co_25_ metallic glass exhibits a pronounced slow β relaxation process. In parallel, strain-rate jump experiments around the glass transition temperature were performed in a wide range of strain rate ranges. The apparent viscosity shows a strong dependence on temperature and strain rate, which reflects the transition from non-Newtonian to Newtonian flow. At low strain or high temperature, a transition was observed from a non-Newtonian viscous flow to Newtonian viscous flow. It was found that the activation volume during plastic deformation of La_30_Ce_30_Al_15_Co_25_ metallic glass is higher than that of other metallic glasses. Higher values of activation volume in La_30_Ce_30_Al_15_Co_25_ metallic glass may be attributed to existence of a pronounced slow β relaxation. It is reasonable to conclude that slow β relaxation in La_30_Ce_30_Al_15_Co_25_ metallic glass corresponds to the “soft” regions (structural heterogeneities) in metallic glass.

## 1. Introduction

Over the past few decades, metallic glasses have attracted a great deal of interest in researchers due to their unique disordered structure. Unlike their crystalline counterparts, metallic glasses do not possess defects like grain boundaries and dislocations [1,2,3,4,5,6,7]. Therefore, they show specific mechanical, chemical and physical properties, such as high strength, high hardness and good corrosion resistance [1,4,8,9,10,11]. As a consequence, metallic glasses are great functional and structural materials from a point of practical application. However, brittle fractures at room temperature severely restrict their application as an engineering material [12]. It is important to tune the plasticity of metallic glasses. Interestingly, when the deformation temperature approaches the glass transition temperature T_g_, metallic glasses usually show excellent thermoplastic capacity [13,14].

It is well documented that amorphous materials stay in a metastable state of energy. Structural relaxation occurs in amorphous materials over time, which is an essential and important feature of these materials. It is believed that there are two typical relaxation processes, i.e., the main α relaxation and the secondary β relaxation, in metallic glasses [8,15,16]. On the one hand, investigations have proven that β relaxation is a reversible process, which is linked to local atomic or molecular movement in amorphous materials [17], while on the other hand β relaxation is an irreversible relaxation, which is related to the dynamic glass transition phenomenon of amorphous materials [18]. Importantly, recent studies have shown that β relaxation may be associated with the plasticity of metallic glasses [17,19].

The plastic deformation of metallic glasses can be divided into two types: homogeneous deformation and inhomogeneous deformation. It has been reported that the mechanical deformation behavior of metallic glasses is sensitive to the temperature and strain rate [20,21]. Similarly, the effects of strain on the plastic deformation of polymers have also been studied, and related mechanisms have been discussed in previous studies [22,23]. Deformation is heterogeneous at low temperatures, which is accompanied by the nucleation and quick propagation of a single shear band, with a brittle fracture then occurring in metallic glasses [24]. At high temperatures (T > 0.8 T_g_), the deformation is homogeneous, and metallic glasses show excellent plasticity [21,25,26]. Previous investigation suggested that the correlation between strength and temperature can be explained by the relaxation activation mechanism [27,28]. Some literature has reported that the transition from Newtonian to non-Newtonian rheology will occur with changes in the temperature and strain rate [21,26,29]. The variation of apparent viscosity is often used as an important indicator of the plastic deformation of metallic glasses. Many theories or models have been proposed to explain the deformation behavior of metallic glasses. The shear transformation zones (STZs) model presumes that atomic clusters undergo torsional deformation under external shear stress [30], whereas the free volume model assumes that plastic deformation is related to the generation and disappearance of free volumes [31].

In the current study, we chose La_30_Ce_30_Al_15_Co_25_ (at. %) metallic glass as a model alloy, due to the LaCe-based metallic glass showing a pronounced slow β relaxation based on the dynamic mechanical analysis [32]. Dynamic mechanical behavior and deformation at high temperatures in LaCe-based metallic glasses have been investigated. The deformation experimental data were analyzed in the framework of the physical models.

## 2. Experimental Procedure

A master alloy of a typical La_30_Ce_30_Al_15_Co_25_ (at. %) metallic glass was prepared by the arc melting method. In order to ensure the homogeneity of the chemical composition of the model alloy, the master alloy was melted at least six times. The metallic glass ribbon used in the current research was prepared by a single roll melt spinning technique in an argon atmosphere. The amorphous nature of the model alloy was determined by X-ray diffraction (XRD, D8 ADVANCE Bruker AXS Gmbh, Karlsruhe, Germany) using Cu Kα radiation at room temperature. The glass transition temperature and onset temperature of crystallization are determined by the differential scanning calorimetry (DSC) technique (commercial instrument NETZCH 404, Bavaria, Germany) with a heating rate of 20 K/min in a nitrogen atmosphere.

The dynamic mechanical behavior of La_30_Ce_30_Al_15_Co_25_ metallic glass was carried out in a commercial dynamic mechanical analysis (DMA, TA Q800, USA) in a tensile mode. The DMA experiment was performed at a fixed frequency of 1 Hz, with a constant heating rate of 2 K/min. The sample was subjected to a periodic sinusoidal strain, and its response stress was simultaneously measured. The complex form of modulus $E^{*}=E^{\prime }+iE^{\prime \prime }$ can be obtained, where $E^{\prime \prime }$ is the loss modulus and $E^{\prime }$ is the storage modulus.

High-temperature deformation experiments on La_30_Ce_30_Al_15_Co_25_ metallic glass ribbon were conducted in a film tensile mode in DMA Q800 at 435, 440, 445 and 450 K, respectively. Initially, La_30_Ce_30_Al_15_Co_25_ metallic glass ribbon was heated to the target temperature, at a rate of 20 K/min. The sample was then kept at the testing temperature for 5 min to ensure thermal equilibration prior to the tensile experiments. The strain rate ranged from 5 × 10^−5^ s^−1^ to 2.5 × 10^−4^ s^−1^.

## 3. Results and Discussion

**Figure 1:**
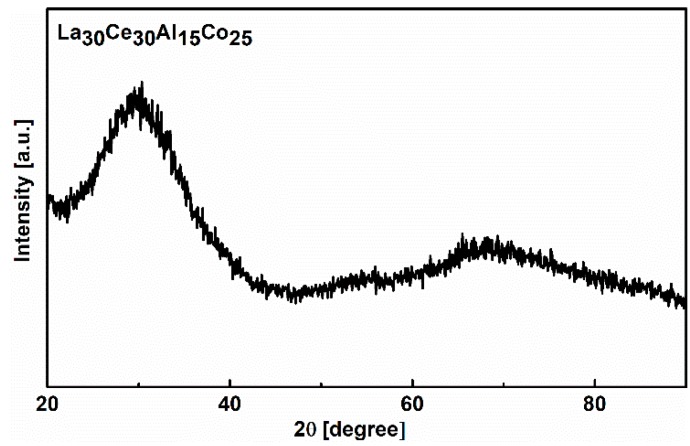
XRD pattern of the as-cast La_30_Ce_30_Al_15_Co_25_ metallic glass.

The glass nature of the model alloy was verified prior to the experiment. Figure 1 displays the XRD pattern of as-cast La_30_Ce_30_Al_15_Co_25_ metallic glass ribbon. No crystalline peak was detected, which confirms the amorphous nature of the model alloy.

**Figure 2:**
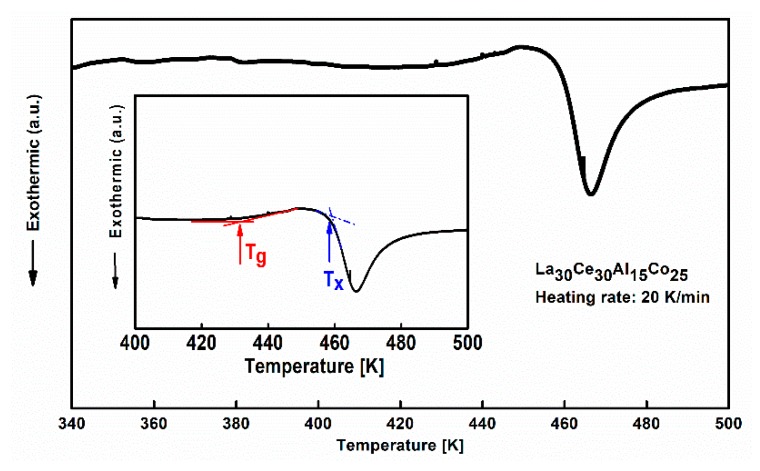
DSC curve of La_30_Ce_30_Al_15_Co_25_ metallic glass (heating rate: 20 K/min). Inset is the definition of the glass transition temperature T_g_ and the onset temperature of crystallization T_x_.

**Figure 3:**
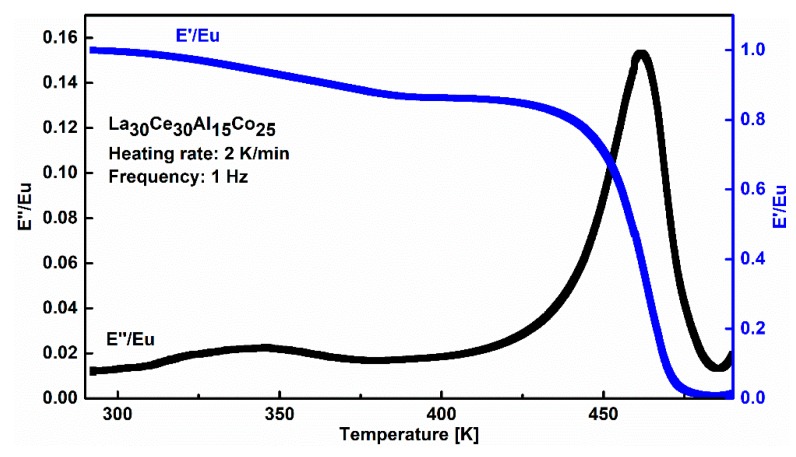
Evolution of the normalized storage modulus and loss modulus with temperature of La_30_Ce_30_Al_15_Co_25_ metallic glass (the driving frequency is 1 Hz and the heating rate is 2 K/min). Eu is the unrelaxed modulus, which is assumed as the storage modulus at an ambient temperature.

In order to explore the thermal characteristics of La_30_Ce_30_Al_15_Co_25_ metallic glass, the DSC measurement was performed at a heating rate of 20 K/min. The glass transition temperature T_g_ and onset temperature of crystallization T_x_ were determined by ASTM-E1356. As shown in Figure 2, the glass transition temperature T_g_ is 430 ± 2 K, and the onset temperature of crystallization T_x_ is 460 ± 2 K. Figure 3 shows the temperature dependence of the normalized storage modulus and loss modulus of La_30_Ce_30_Al_15_Co_25_ metallic glass. Three different regions can be observed: (1) At a low temperature region (from room temperature to 400 K), a peak corresponding to slow β relaxation around 348 K can be observed, which is considered to be related to the structure inhomogeneity of metallic glass in previous research [19]. However, many metallic glasses show only an “excess wings” or “shoulders” based on the loss modulus [15]; (2) As the temperature increases from 400 K and 480 K, which mainly corresponds to the supercooled liquid region of La_30_Ce_30_Al_15_Co_25_ metallic glasses. Clearly, the storage modulus drops drastically while the loss modulus increases rapidly up to its maximum, which corresponds to the main α relaxation. The magnitude of α relaxation is much higher than β relaxation, which can be attributed to the large-scale movement of atoms [1,16]; (3) When the temperature surpasses 480 K, wherein the sample undergoes an irreversible structural transformation, with the storage modulus again increasing due to the introduction of the crystalline phase with the temperature. These results are in good agreement with the previous study [16,32].

**Figure 4:**
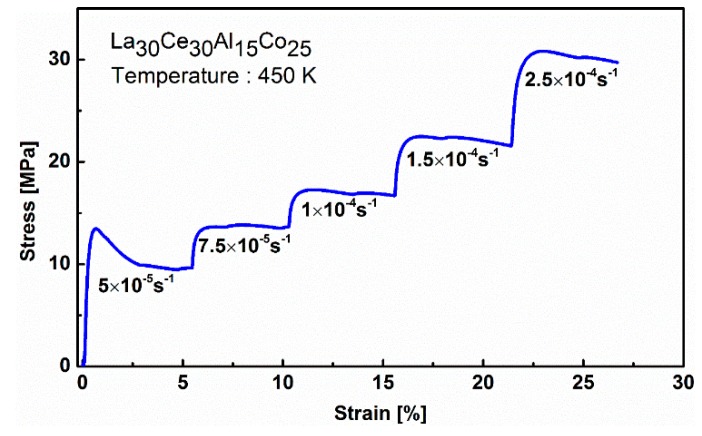
A typical tensile strain-stress curve of La_30_Ce_30_Al_15_Co_25_ metallic glass ribbon during strain-rate jump experiments at a given temperature of 450 K.

In order to further study the mechanical behavior of LaCe-based metallic glass at high temperatures, tensile strain-rate jump tests were carried out, with the strain rates ranging from 5 × 10^−5^ s^−1^ to 2.5 × 10^−4^ s^−1^. Typically, the deformation behaviors of metallic glasses depend on both the temperatures and applied strain rates [20,21,33]. A typical tensile strain-stress curve of La_30_Ce_30_Al_15_Co_25_ metallic glass ribbon during a strain-rate jump test at 450 K was shown in Figure 4.

**Figure 5:**
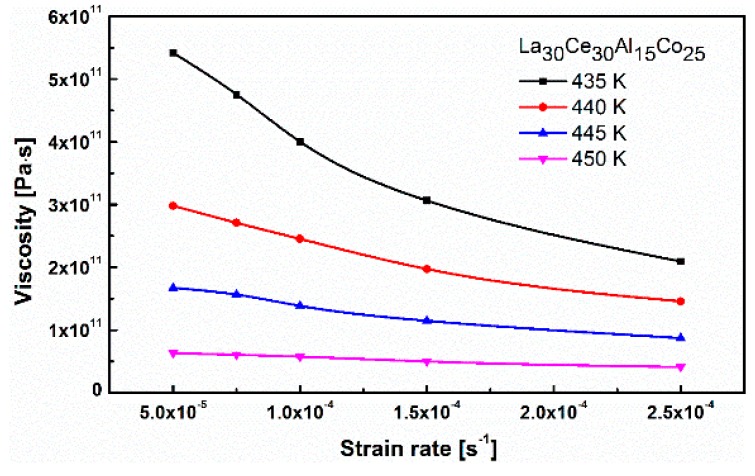
The apparent viscosities of La_30_Ce_30_Al_15_Co_25_ metallic glass ribbon as functions of the strain rates at different temperatures.

It is clear to see that the steady flow stress increases by increasing the strain rate. At a given strain rate, the steady flow stress will drop by increasing the measurement of the temperature. According to the strain-rate jump tests, the apparent viscosity can be calculated as [34]

(1) $$\eta =\frac{\sigma_{f}}{3\dot{\varepsilon }}$$

where $\sigma_{f}$ is the steady state flow stress and $\dot{\varepsilon }$ is the applied strain rate. Figure 5 shows the effect of the temperature and strain rate on the apparent viscosity of La_30_Ce_30_Al_15_Co_25_ metallic glass. It can be found that the apparent viscosity gradually decreases by increasing the strain rate at a lower temperature. When the temperature is below 450 K, the viscosity changes significantly with the strain rate. The current research is in accordance with the non-Newtonian behavior rheological properties found in previous studies [21,35]. On the other hand, when the temperature is above 450 K, the deformation turns into Newtonian behavior. Obviously, the apparent viscosity is not sensitive to the strain rate [33,36].

The high-temperature tensile deformation of metallic glasses is usually described as [31]

(2) $$\dot{\varepsilon }=2c_{f}v_{D}\exp\left(-\frac{\Delta G^{m}}{\mathrm{KT}}\right)\sin h\left(\frac{\sigma V}{2\sqrt{3}\mathrm{KT}}\right)$$

where *K* is the Boltzmann constant, T is the temperature, $v_{D}$ is the Debby frequency, $\Delta G^{m}$ is the activation energy for defect migration and $c_{f}$ is the concentration of flow defect. The hyperbolic sine term takes the effect of the applied stress on the energy barrier into account. V is the activation volume, defined as the volume corresponding to each elementary defect jump. The energy barrier will be affected by the activation volume when the flow stress increases. Up to now, there is no perfect explanation for the defects in high-temperature deformation. It should be noted that the defect concentration is closely related to the change of free volume [31]. Equation (2) can also be simplified to the following formula [37]

(3) $$\dot{\varepsilon }={\dot{\varepsilon }}_{0}\sin h\left(\frac{\sigma V}{2\sqrt{3}\mathrm{KT}}\right)$$

with

(4) $${\dot{\varepsilon }}_{0}\left(c_{f}\right)=2c_{f}v_{D}\exp\left(-\frac{\Delta G^{m}}{\mathrm{KT}}\right)$$

**Figure 6:**
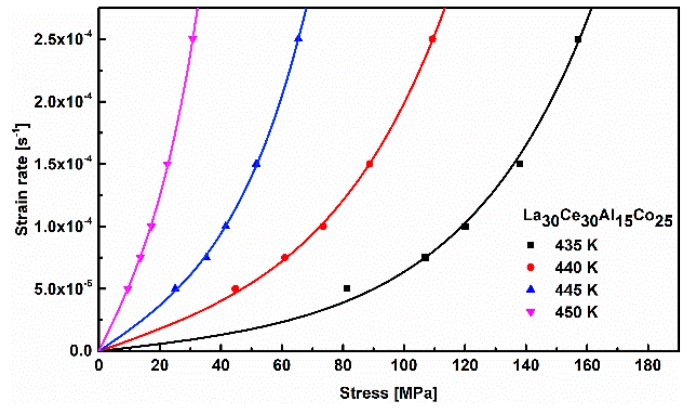
Correlation between the flow stress and strain rate of La_30_Ce_30_Al_15_Co_25_ metallic glass at different temperatures. The solid line is the fitting curve.

**Figure 7:**
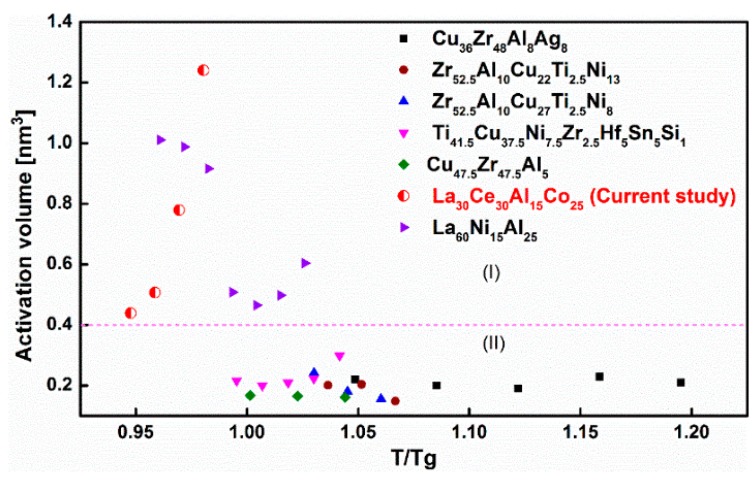
The activation volume varies with testing temperature for typical metallic glasses in high-temperature deformation (testing temperature around the glass transition temperature T_g_). (I) La-based metallic glasses with pronounced β relaxation (II) other metallic glasses without obvious β relaxation. The activation volume data obtained in the present research and the previous literatures [37,38,45].

Figure 6 shows the correlation between the flow stress and applied strain rate at various temperatures. The activation volume V and frequency factor ${\dot{\varepsilon }}_{0}$ can be determined (as shown in Table 1). Based on Equation (2), the values of activation volume V and the frequency factor ${\dot{\varepsilon }}_{0}$ can be derived for La_30_Ce_30_Al_15_Co_25_ metallic glass. It can be clearly seen from Table 1 that the activation volume increases with the temperature. The activation can be defined as V = $\gamma_{0}\Omega_{f}$, where $\gamma_{0}$ represents the local shear strain and $\Omega_{f}$ is the volume of the involved defect. $\gamma_{0}$ is often taken as 0.125 in previous studies [37]. Taking into account the values of $\gamma_{0}$, the volume of the LaCe-base metallic glass flow defect volume $\Omega_{f}$ can be determined to be about 4.1 nm^3^. In addition, according to the activation volume data shown in Figure 7, the value $\Omega_{f}$ of several typical metallic glasses ranges from 0.8 nm^3^ to 2.24 nm^3^. LaCe-based metallic glass involves greater flow defects in high-temperature deformation than that of other typical metallic glasses. This result is consistent with the previous report on La_65_Ni_15_Al_25_ metallic glass [38].

In Figure 7, it is clearly observed that the activation volume of La_30_Ce_30_Al_15_Co_25_ metallic glass at high-temperature deformation is more than 0.4 nm^3^. The average volume of atoms values Ω is considered to be 0.013 nm^3^ [39]. It is reasonable to conclude that the activation volume for La_30_Ce_30_Al_15_Co_25_ metallic glass corresponds to about 40 atomic volumes. Interestingly, Figure 7 can be divided into two regions: Region (I) is the activation volume of La_30_Ce_30_Al_15_Co_25_ and La_65_Ni_15_Al_25_ [38] metallic glasses, which, according to the DMA results, exhibit pronounced slow β relaxation; and Region (II), in which metallic glasses do not exhibit distinct slow β relaxation with a smaller activation volume, such as Cu-based, Zr-based and Ti-based metallic glasses, whose activation volume involved in the local motion of the atom is approximately 20 atomic volumes. A larger activation volume is linked to more atoms being activated during the plastic deformation. In general, a larger activation volume perhaps shows a high concentration of defects to some extent.

**Figure 8:**
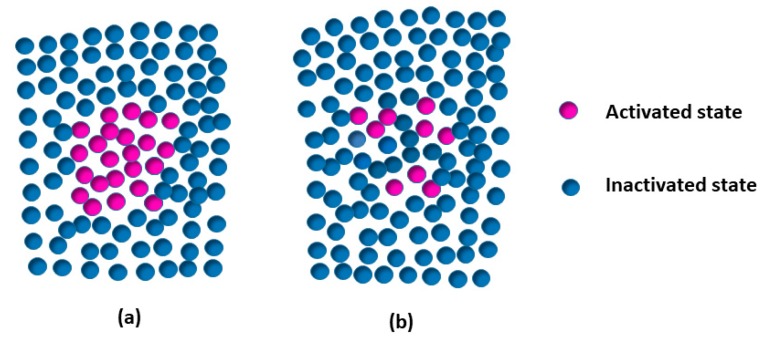
The schematic diagram of activation volumes in metallic glasses at the high-temperature deformation: (**a**) Metallic glasses show a pronounced β relaxation; and (**b**) β relaxation is not evident (i.e., excess wing or shoulder) in metallic glasses.

The microstructure heterogeneity of amorphous alloys has been confirmed by electron microscopy or simulations. It is believed that β relaxation is related to structural heterogeneity. In parallel, the activation of β relaxation is considered to be an indicator of achieving plastic properties [40,41]. In addition, β relaxation is usually considered to be a partial migration of local atoms and the plastic units, i.e., STZs, which are involved in the motions of dozens of atoms. The activation energy of β relaxation and STZs have been confirmed to be almost the same [42]. Several models have been proposed to explain the structural heterogeneity of metallic glasses with the help of experiment or theory. The flow units model suspects that deformation in amorphous alloys is borne by the flow units [43]. Perez proposed the “quasi-point defects” (QPD) model, which assumes that there are fluctuations of entropy or enthalpy in amorphous solids [44]. It is well accepted that β relaxation is associated with structural heterogeneity in metallic glasses [8,16]. Generally, it is believed that the structural heterogeneity of metallic glasses includes a soft “liquid-like” region and a hard “solid-like” region [8,42]. β relaxation probably originated from the “soft” region [38,41], where the atoms are more loosely arranged and easier to move. Therefore, more atoms could be activated under mechanical or thermal stimulation in LaCe-based metallic glasses. Figure 8a,b show the activation volume diagram for the metallic glasses which have pronounced β relaxation or not, respectively. From Figure 8, it can be seen that more atoms could be activated in metallic glasses with a pronounced β relaxation process. As evidenced by Figure 7, two La-based metallic glasses with pronounced β relaxation in area (I) possess more activation volumes than other metallic glasses without obvious β relaxation in area (II). This indicates that metallic glasses with pronounced β relaxation have more atoms involved in their motion during a steady flow at a high temperature, which is in agreement with the above analysis.

In the Newtonian flow region, it is assumed that the flow defects concentration $c_{f}$ is related to the equilibrium volume of a Newtonian state. Therefore, Newtonian equilibrium viscosity η_N_ can be determined by:

(5) $$\eta_{N}=\frac{2\sqrt{3}\mathrm{KT}}{3\dot{\varepsilon_{0}}V}$$

**Figure 9:**
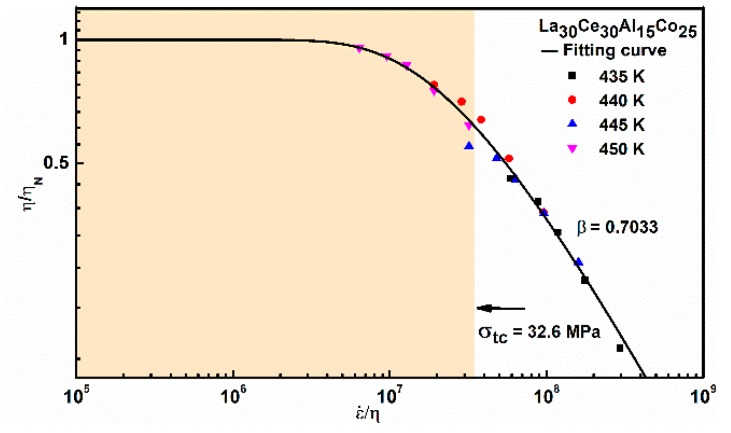
A master curve of La_30_Ce_30_Al_15_Co_25_ metallic glass at high temperature tensile deformation between normalized viscosity η/η_N_ and $\dot{\varepsilon }\eta$_N._ The solid line is fitted by Equation (6).

Newtonian equilibrium viscosity η_N_ at different temperatures can be obtained from the experimental data. The values are 1.18 × 10^12^ Pa s, 3.84 × 10^12^ Pa s, 3.04 × 10^12^ Pa s and 6.63 × 10^10^ Pa s for T = 435 K, 440 K, 445 K and 450 K, respectively. In addition, the viscosity master curve is an approach to discuss the deformation of metallic glass. Figure 9 shows a master curve between the normalized viscosity η/η_N_ and $\dot{\varepsilon }\eta$_N_. Viscosity at different temperatures was normalized with the corresponding Newtonian viscosity, and the main curve was obtained by shifting with 440 K as the reference temperature. The master curve can be well described by a stretched exponential function, the Kohlraush–Williams–Watts (KWW) model, based on a previous investigation [29]

(6) $$\frac{\eta }{\eta_{N}}=1-\exp\left[-{\left(\frac{\sigma_{\mathrm{tc}}}{{\dot{\varepsilon }\eta }_{N}}\right)}^{\beta_{\mathrm{kww}}}\right]$$

where $\sigma_{\mathrm{tc}}$ and $\beta_{\mathrm{kww}}$ are fitting parameters. $\sigma_{\mathrm{tc}}$ can be regarded as the characteristic stress of non-Newtonian rheology to Newtonian rheological transformation. Usually, $\beta_{\mathrm{kww}}$ ranges from 0 to 1. It can be seen from Figure 9, and experimental data can be well described by Equation (6). The value of characteristic stress $\sigma_{\mathrm{tc}}=32.6\;\mathrm{MPa}$ is related to a transformation from non-Newtonian to Newtonian behavior. $\beta_{\mathrm{kww}}=0.7$ is in good accordance with the values of $\beta_{\mathrm{kww}}$ in previous research results: Zr_41.2_Ti_13.8_Cu_12.5_Ni_10_Be_22.5_ [46], La_55_Al_25_Ni_20_ [47] and Zr_65_Cu_18_Ni_7_Al_10_ [29]. It could be concluded that a smaller $\beta_{\mathrm{kww}}$ value means a larger deviation from Newtonian flow.

**Figure 10:**
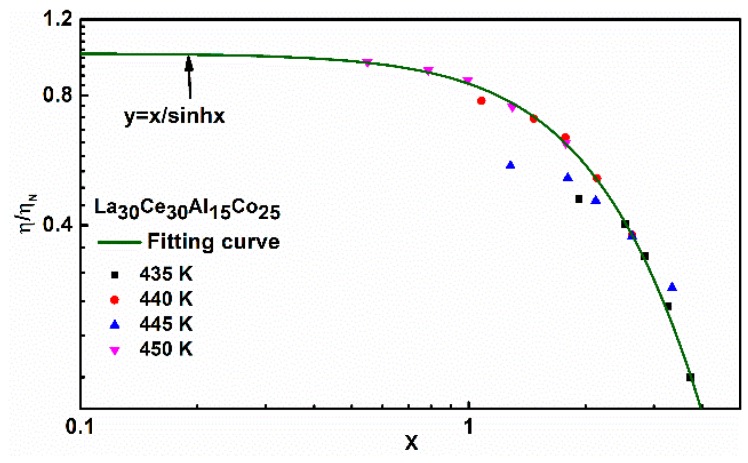
Master curve between normalized viscosity η/η_N_ and X at different temperatures, where X = $\sigma V/\left(2\sqrt{3}\mathrm{KT}\right)$. The solid line is fitted by Equation (7).

According to previous investigations, the master curve of viscosity can be described as [48]

(7) $$\frac{\eta }{\eta_{N}}=\frac{\sigma V/\left(2\sqrt{3}\mathrm{KT}\right)}{\sin h\left[\sigma V/\left(2\sqrt{3}\mathrm{KT}\right)\right]}=\frac{X}{\sin\mathrm{hX}}$$

where X = $\sigma V/\left(2\sqrt{3}\mathrm{KT}\right)$. Figure 10 shows the master curve between the normalized viscosity η/η_N_ and X at different temperatures. It should be noted that Newtonian flow occurs when the normalized viscosity tends to a constant. With the variation of temperature and strain rate, there will be a transition between Newtonian and non-Newtonian flow.

## 4. Conclusions

High-temperature deformation behavior and the dynamic mechanical relaxation process of La_30_Ce_30_Al_15_Co_25_ metallic glass were investigated in the current work. Compared with other metallic glasses, La_30_Ce_30_Al_15_Co_25_ metallic glass shows a pronounced slow β relaxation, based on the DMA results. We found that the activation volumes during the steady flow of La-based and La_30_Ce_30_Al_15_Co_25_ metallic glasses with a pronounced β relaxation are much higher than that of other typical metallic glasses without obvious β relaxation. This phenomenon can be attributed to the same origin of the β relaxation of metallic glasses, i.e., the structural heterogeneity.

## Figures and Tables

**Table 1:** The activation volume and frequency factor ${\dot{\varepsilon }}_{0}$ of La_30_Ce_30_Al_15_Co_25_ metallic glass at different temperatures are calculated from experimental data.

Temperature (K)	Activation Volume (nm^3^)	Frequency Factor ${\dot{\varepsilon }}_{0}$ (S^−1^)
435	0.439	1.20 × 10^−5^
440	0.507	3.59 × 10^−5^
445	0.779	4.59 × 10^−5^
450	1.240	8.70 × 10^−5^
